# Comparative application of MAFLD and MASLD diagnostic criteria on NAFLD patients: insights from a single-center cohort

**DOI:** 10.1007/s10238-024-01553-3

**Published:** 2025-01-14

**Authors:** Maha Elsabaawy, Madiha Naguib, Ahmed Abuamer, Ahmed Shaban

**Affiliations:** Department of Hepatology and Gastroenterology, National Liver Institute, Menoufia University, Shebeen Elkoom, Menoufia, Egypt

**Keywords:** NAFLD, MAFLD, MASLD, Fibrosis, Hepatic steatosis, Cardiovascular risk

## Abstract

The diagnostic criteria for Metabolic Dysfunction-Associated Fatty Liver Disease (MAFLD) and Metabolic Associated Steatotic Liver Disease (MASLD) aim to refine the classification of fatty liver diseases previously grouped under Non-Alcoholic Fatty Liver Disease (NAFLD). This study evaluates the applicability of the MAFLD and MASLD frameworks in NAFLD patients, exploring their clinical utility in identifying high-risk patients. A total of 369 NAFLD patients were assessed using MAFLD and MASLD diagnostic criteria. Baseline characteristics, metabolic profiles, hepatic fibrosis, and cardiovascular risks were compared across the groups. Among NAFLD patients, 97.55% (*n* = 359) met MASLD criteria, and 97.01% (*n* = 357) fulfilled MAFLD criteria. Both frameworks MAFLD and MASLD captured overlapping populations, with MASLD encompassing slightly more cases. No significant differences were observed in metabolic risk factors, fibrosis indices (APRI, FIB-4, NAFLD fibrosis score), or cardiovascular risk (10-year ASCVD score). A small subset of lean NAFLD patients (10 cases) with distinct profiles remained uncategorized by either framework. Pure NAFLD cases (*n* = 10) were with mild insulin resistance (HOMA-IR: 3.07 ± 0.33) and slightly elevated LDL (102.5 ± 42.87 mg/dL), while fibrosis indices indicated low fibrosis risk. Steatosis indices supported the diagnosis of early-stage NAFLD with preserved liver function. These patients do not meet the criteria for inclusion in the MAFLD or MASLD frameworks, highlighting a gap in the current diagnostic systems. MAFLD and MASLD criteria align closely with NAFLD in capturing patients with metabolic risk with MASLD-enhanced inclusivity. Further refinement is required to address heterogeneity, particularly in lean NAFLD patients. Hypertension prevalence was comparable (17.4% in NAFLD, 18.2% in MAFLD, 17.8% in MASLD; *p* = 0.960), as was diabetes mellitus (36.7%, 37.8%, and 37.6%, respectively; *p* = 0.945). Body mass index was also similar across groups, with medians of 33.25, 33.6, and 33.4 kg/m^2^ (*p* = 0.731). Non-invasive markers of hepatic fibrosis, including APRI, FIB-4, and NAFLD fibrosis scores, did not differ significantly, with median FIB-4 scores around 1.05 (*p* = 0.953). Similarly, were the results of hepatic steatosis index and ASCVD score.

## Introduction

The landscape of chronic liver disease in Egypt has drastically changed over the past few decades, with the decreasing prevalence of viral hepatitis and increasing prevalence of fatty liver disease (FLD) [1]. FLD in the Middle East/North Africa region could be of particular concern because of cumulative reports describing exceptionally high prevalence rates of the disease, reaching up to 56% of the population in Egypt. More awareness and education with affordable risk stratification tools are necessary to combat the growing disease burden in the region Non-alcoholic steatohepatitis (NASH) was first recognized by Ludwig and colleagues in 1980 and was characterized by liver steatosis, hepatocyte injury, liver inflammation, and fibrosis. Subsequently, the term non-alcoholic fatty liver disease (NAFLD) was used in 1986 to describe the histological spectrum of steatosis to steatohepatitis with its subtypes NAFL and NASH [2].

The term NAFLD literally refers to non-alcohol related hepatopathy and does not adequately correlate with metabolic dysfunction and related cardiovascular risks. Therefore, researchers and scientific societies have moved towards changing the terminology [3]. In 2020, a panel of international experts, mostly from the Asian-Pacific region, proposed to change its name to “metabolic dysfunction-associated fatty liver disease” or MAFLD [4–6].

Accordingly, MAFLD was linked to the presence of metabolic abnormalities, irrespective of comorbidities and other causes of chronic liver disease. In addition, the stigma of “fatty” remains in the nomenclature of MAFLD [7].

Three years later, the Delphi consensus statement, led by the American Association for the Study of Liver Diseases (AASLD), the European Association for the Study of the Liver (EASL), and the Association Latinoamericana parael Estudio del Hígado (ALEH) and co-authored by 53 experts from around the globe, proposed adopting the term “steatotic liver disease” (SLD) as an integrative description of the various etiologies of steatosis [8].

The term metabolic dysfunction associated steatotic liver disease (MASLD) has been proposed, and it can be diagnosed based on a patient meeting one of five cardiovascular risk factors, unlike MAFLD, which required that patients meet two of seven parameters of metabolic dysfunction. Among them, patients who meet both MASLD and alcohol-related fatty liver disease (ALD) criteria are categorized as having MetALD. Clarifying the differences of clinical and histological features facing different FLD nomenclatures provides reference for developing therapeutic strategies [9].

These changes are expected to improve the awareness of practitioners and their patients, remove stigmatization by avoiding inappropriate labels, facilitate further research, and open the door to personalized therapeutic approaches. This is a lot of expectations and there is more work to do [4].

We were aiming to enhance the understanding of the clinical application and validity of the updated nomenclatures on NAFLD patients and provide helpful knowledge into its potential impact on patients.

## Methods

### Patients

This cross-sectional study recruited 368 consecutive patients from the fatty liver clinic at the National Liver Institute, Menoufia University, Egypt, between January 2023 and January 2024. The study was approved by the Ethics Committee of the National Liver Institute and was performed in accordance with the 1975 Declaration of Helsinki. Included patients aged 18 years and older who met the diagnosis of Fatty liver, now named steatotic liver disease (SLD), verified through abdominal ultrasonography after exclusion of viral hepatitis, drug-induced liver disease, autoimmune hepatitis, or metabolic/genetic liver diseases. Following the recommended diagnostic criteria, the patients were classified as NAFLD, MAFLD, and MASLD.

### Definitions of different FLDs

**NAFLD** was defined as the presence of hepatic steatosis without excessive alcohol consumption (≥ 20 g/d for females and ≥ 30 g/d for males) and/or other liver diseases [10].

**MAFLD** is based on the presence of hepatic steatosis and at least one of these conditions: type 2 diabetes mellitus (T2DM), obesity, and metabolic dysregulation. Metabolic dysregulation is defined as the presence of at least two metabolic risk abnormalities between: waist circumference (WC) ≥ 102cm for men/88cm for women, blood pressure ≥ 130/85 mmHg or antihypertensive medication, plasma Triglycerides (TG) ≥ 150 mg/dl or triglycerides lowering medication, plasma high-density lipoprotein cholesterol (HDL-C) < 40 mg/dl for men and < 50 mg/dl for women or lipid-lowering medication, prediabetes (fasting plasma glucose levels between 100 and 125 mg/dl or 2 h post-load glucose levels between 140 and 199 mg/dl or glycosylated hemoglobin (HbA1c) between 5.7 and 6.4%, Homeostatic Model Assessment for Insulin Resistance (HOMA-IR) score ≥ 2.5, or high-sensitivity C-reactive protein levels > 2 mg/L [11].

**MASLD** was defined as hepatic steatosis combined with at least 1 of the 5 cardiometabolic adult criteria and without other discernible causes for hepatic steatosis. The five cardiometabolic criteria include (i) BMI ≥ 25kg/m2 or WC > 102/88 cm in men and women; (ii) fasting glucose ≥ 100 mg/dL, 2-h post-load glucose levels ≥ 140 mg/dL, HbA1c ≥ 5.7%, type 2 diabetes mellitus (DM), or treatment for type 2 DM; (iii) blood pressure ≥ 130/85 mmHg or specific antihypertensive drug treatment; (iv) plasma TG ≥ 150 mg/dL or lipid-lowering treatment; and (v) plasma HDL-C ≤ 40 mg/dL for men and ≤ 50 mg/dL for women or lipid-lowering treatment [7].

### Clinical examination and laboratory biochemical measurements

Blood pressure, weight, height, waist and waist-hip circumferences were measured and BMI was calculated as body weight in kilograms divided by height in meters squared. Complete blood count (CBC), serum albumin, total bilirubin, aspartate aminotransferase (AST), alanine transaminase (ALT), alkaline phosphatase (ALP), γ-glutamyl transferase (GGT), prothrombin time (PT), international normalized ratio (INR), blood urea nitrogen (BUN), serum creatinine, serum uric acid, fasting plasma glucose (FPG), fasting plasma insulin, HbA1c, TG, TC, low-density lipoprotein cholesterol (LDL-C), and HDL-C were performed by standard laboratory methods and HOMA-IR was calculated as fasting insulin (μU/mL) × fasting glucose (mmol/L)/22.5.

The severity of liver fibrosis was determined by previously confirmed liver fibrosis prediction models: fibrosis-4 index (**FIB4**), NAFLD fibrosis score (**NFS**) and Aspartate aminotransferase to platelet ratio index (**APRI**) [11].

Also, two non-invasive models were used to assess liver steatosis in this study: hepatic steatosis index (**HSI**) and Fatty Liver Index (**FLI**) [12].

The pooled 10-year ASCVD risk estimation equation from the 2013 American College of Cardiology/American Heart Association guidelines was used. As described by prior studies and available to review on online calculator (https://tools.acc.org/ldl/ascvd_risk_estimator/index.html#!/calulate/estimator/). [13, 14].

### Statistical analysis

Data were fed to the computer and analyzed using IBM SPSS software package version 20.0. (Armonk, NY: IBM Corp). Categorical data were represented as numbers (n) and percentages (%). As to demographic parameters, the intergroup differences in percentages were examined by the chi-square test. For continuous data, they were tested for normality by the Kolmogorov–Smirnov test. Numeric variables are expressed as means ± standard deviation (SD) for parameters with normal distributions and as medians (interquartile ranges) for parameters with abnormal distributions. Kruskal Wallis test was used to compare different groups and followed by Post Hoc test (Dunn's for multiple comparisons test) for pairwise comparison.

## Results

### Study subjects

A total of 369 patients with fatty liver were included, with a mean age of 47.01 ± 10.84 years. The majority were females (65.1%), their mean BMI was 34.13 ± 6.29 kg/m2, 36.7% had diabetes mellitus (DM), and 17.7% were hypertensive. The patients were classified as NAFLD, MAFLD, and MASLD following the recommended diagnostic criteria. 100% (n = 369) was NAFLD, 97.55% (n = 359) was MASLD while 97.01% (n = 357) was MAFLD (Table 1).

**Table 1:** Baseline characteristics of different SLD (n = 369)

	**NAFLD** **(n = 369)**	**MAFLD** **(n = 357)**	**MASLD** **(n = 359)**	**Test of Sig**	**P**
**Gender**					
Male	129 (34.9%)	118 (33.1%)	120 (33.4%)	χ^2^ = 0.270	0.874
Female	240 (65.1%)	239 (66.9%)	239 (66.6%)		
**Age (years)**					
Min. – Max	18.0 – 75.0	18.0 – 75.0	18.0 – 75.0	H = 0.325	0.850
Mean ± SD	47.01 ± 10.84	47.41 ± 10.79	47.31 ± 10.80		
Median (IQR)	45.0 (39.0 – 55.50)	47.0 (39.0 – 56.0)	47.0 (39.0 – 56.0)		
**HTN**	64 (17.4%)	65 (18.2%)	64 (17.8%)	χ^2^ = 0.083	0.960
**DM**	135 (36.7%)	135 (37.8%)	135 (37.6%)	χ^2^ = 0.112	0.945
**BMI (kg/m**^**2**^**)**					
Min. – Max	18.30 – 57.60	18.30 – 57.60	18.30 – 57.60	H = 0.628	0.731
Mean ± SD	34.13 ± 6.29	34.48 ± 6.09	34.36 ± 6.19		
Median (IQR)	33.25 (30 – 37.70)	33.6 (30.50 – 37.9)	33.40(30.30 –37.9)		
**Waist circumference**					
Min. – Max	69.86 – 149.9	69.86 – 149.9	69.86 – 149.9	H = 0.019	0.991
Mean ± SD	113.3 ± 13.14	113.3 ± 13.11	113.1 ± 13.36		
Median (IQR)	113 (105 – 121)	113 (105 – 121)	113 (105 – 121)		
**Waist/hip**					
Min. – Max	0.77 – 1.15	0.77 – 1.15	0.77 – 1.15	H = 0.007	0.997
Mean ± SD	0.95 ± 0.07	0.95 ± 0.07	0.95 ± 0.07		
Median (IQR)	0.95 (0.91 – 0.99)	0.95 (0.91 – 0.99)	0.95 (0.91 – 0.99)		

HTN, hypertension; DM, diabetes mellitus; BMI, body mass index; M, mean; SD, standard deviation; IQR: Inter quartile range, SD: Standard deviation, χ^2^: Chi square test, H: H for Kruskal Wallis test, p: p value for comparing between the three studied groups *: Statistically significant at p ≤ 0.05

### Classification of various SLD

**Fig. 1:**
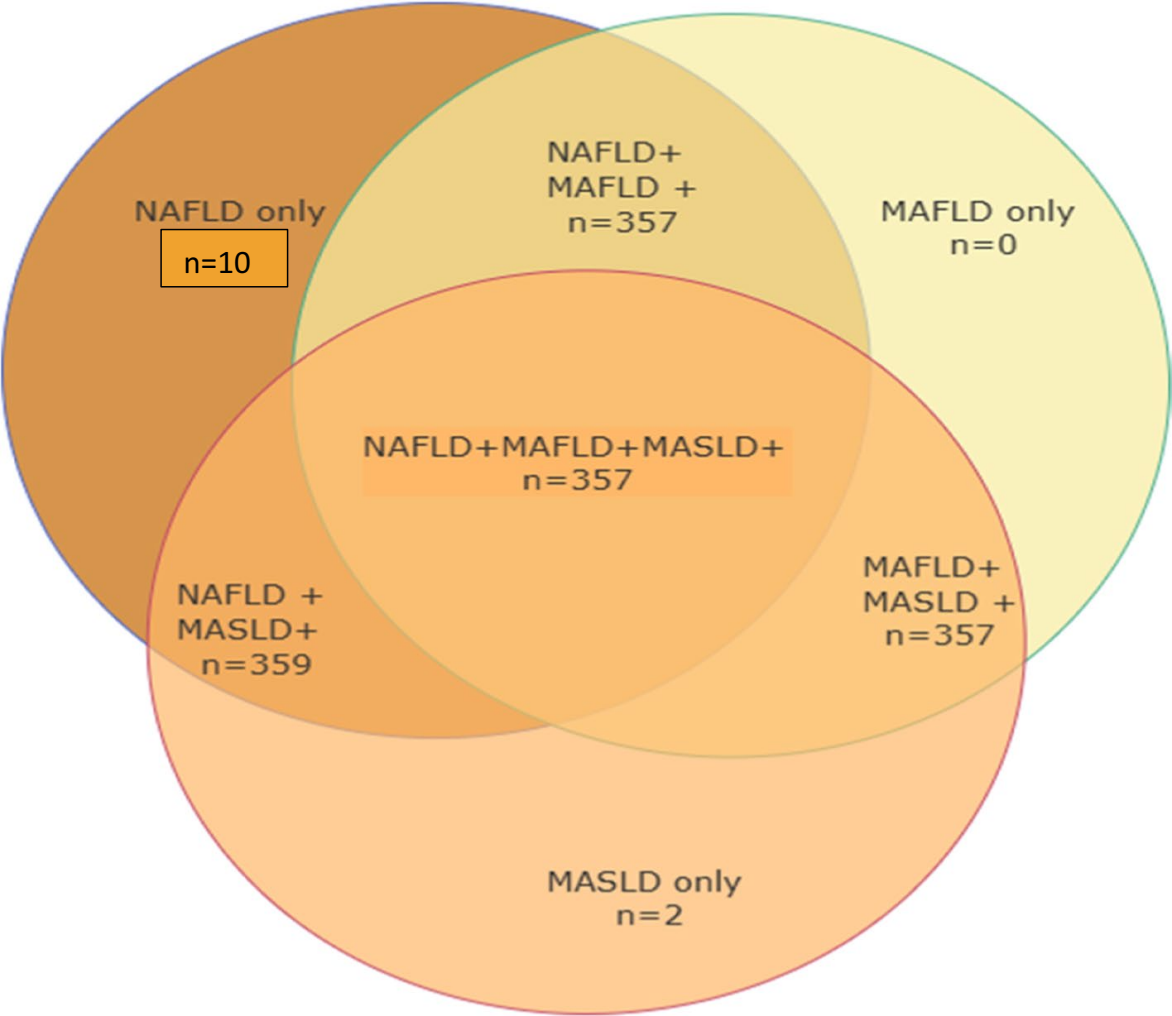
Classification of various SLD

Regarding NAFLD patients, 97.55% (n = 359) were classified to have MASLD (NAFLD + MASLD +), and 97.01% (n = 357) were determined as MAFLD (NAFLD + MAFLD +), 2.7% (n = 10) patients with NAFLD did not meet the MAFLD criteria because they had lean BMI < 25 kg/m2 with fewer than two metabolic risk factors (NAFLD + MAFLD-). 2.2% (n = 8) patients with NAFLD did not meet the MASLD criteria because they had lean BMI < 25 kg/m2 with a lack of any metabolic risk factors (NAFLD + MASLD-). For MAFLD patients, 100% (n = 357) followed NAFLD and MASLD (NAFLD + MASLD + MAFLD +). As to MASLD patients, 100% (n = 359) individuals were by NAFLD, and 99.44% (n = 357) individuals were under MAFLD (Fig. 1).

### Similarities and differences of different groups

Gender distribution was similar among NAFLD (34.9% male), MAFLD (33.1% male), and MASLD (33.4% male) groups (*p* = 0.874). The median age was consistent across groups: 45 years in NAFLD, 47 years in MAFLD, and 47 years in MASLD (*p* = 0.850). Hypertension prevalence was comparable (17.4% in NAFLD, 18.2% in MAFLD, 17.8% in MASLD; p = 0.960), as was diabetes mellitus (36.7%, 37.8%, and 37.6%, respectively; *p* = 0.945). Body mass index (BMI) was also similar across groups, with medians of 33.25, 33.6, and 33.4 kg/m^2^ (*p* = 0.731). Aso, regarding to liver enzymes, total bilirubin, TG, TC, LDL-C, HDL-C, FPG, and HOMA-IR we did not find any significant differences between NAFLD, MAFLD and MASLD (*p* > 0.05) (Table 2).

**Table 2:** laboratory parameters of the study population

	**NAFLD** **(n = 369)**	**MAFLD** **(n = 357)**	**MASLD** **(n = 359)**	**Test of Sig**	**P**
**AST**					
Min. – Max	11 – 130	11 – 130	11 – 130	H = 0.013	0.994
Mean ± SD	36.54 ± 20.55	36.55 ± 20.41	36.51 ± 20.58		
Median (IQR)	31.50 (23 – 42)	31.50 (23 – 42)	31.50 (23 – 42)		
**ALT**					
Min. – Max	11 – 130	11 – 130	11 – 130	H = 0.027	0.987
Mean ± SD	41.69 ± 19.35	41.93 ± 19.54	41.78 ± 19.58		
Median (IQR)	38.10(28.2 – 49.4)	38.70 (28 – 50)	38.50 (28 – 49.90)		
**GGT**					
Min. – Max	3 – 309.9	3 – 309.9	3 – 309.9	H = 0.019	0.991
Mean ± SD	43.53 ± 30.19	43.53 ± 30.51	43.47 ± 30.46		
Median (IQR)	35 (23 – 54)	35 (23 – 54)	35 (22.75 – 53.9)		
**INR**					
Min. – Max	0.89 – 1.20	0.89 – 1.20	0.89 – 1.20	H = 0.033	0.984
Mean ± SD	1.02 ± 0.06	1.02 ± 0.06	1.02 ± 0.06		
Median (IQR)	1.0 (0.99 – 1.04)	1.0 (0.99 – 1.04)	1.0 (0.99 – 1.04)		
**Platelet**					
Min. – Max	92 – 578	92 – 578	92 – 578	H = 0.003	0.999
Mean ± SD	228.4 ± 54.65	228 ± 54.22	228 ± 54.13		
Median (IQR)	221 (192 – 253.50)	221 (192 – 253)	221 (192 – 253)		
**T. cholesterol**					
Min. – Max	90 – 416	90 – 416	90 – 416	H = 0.196	0.907
Mean ± SD	218.6 ± 61.56	220.3 ± 61.32	219.8 ± 61.45		
Median (IQR)	215 (167 – 258)	215 (172 – 258)	215 (171 – 258)		
**HDL**					
Min. – Max	6.70 – 144	6.70 – 144	6.70 – 144	H = 0.014	0.993
Mean ± SD	50.04 ± 21.52	50.26 ± 21.81	50.16 ± 21.77		
Median (IQR)	44 (38.75 – 55)	44 (38.50 – 55)	44 (38.50 – 55)		
**LDL**					
Min. – Max	5.80 – 329.7	5.80 – 329.7	5.80 – 329.7	H = 0.114	0.944
Mean ± SD	137.3 ± 59.99	138.5 ± 60.14	138.2 ± 60.10		
Median (IQR)	129.7(91.70 – 174)	131.2(93.20 –174.1)	131.1(93.10 –174.1)		
**TG**					
Min. – Max	52.00 – 590.0	52.00 – 590.0	52.00 – 590.0	H = 0.127	0.938
Mean ± SD	156.1 ± 74.69	157.7 ± 75.23	157.3 ± 75.18		
Median (IQR)	141.0(100.0 – 180.0)	146.4(101.0 – 181.3)	146.2(100.5 – 180.7)		
**HbA1C**					
Min. – Max	4.20 – 14	4.20 – 14	4.20 – 14	H = 0.145	0.930
Mean ± SD	6.01 ± 1.48	6.04 ± 1.49	6.03 ± 1.49		
Median (IQR)	5.50 (5 – 6.50)	5.60 (5 – 6.50)	5.60 (5 – 6.50)		
**HOMA-IR**					
Min. – Max	0.34 – 17.01	0.34 – 17.01	0.34 – 17.01	H = 0.003	0.999
Mean ± SD	3.41 ± 2.42	3.42 ± 2.45	3.42 ± 2.45		
Median (IQR)	2.98 (1.71 – 4.50)	2.97 (1.69 – 4.56)	2.94 (1.69 – 4.55)		

AST, aspartate aminotransferase; ALT, alanine aminotransferase; GGT, gamma-glutamyl transferase; INR, international normalized ratio; HDL, high-density lipoprotein; LDL, low-density lipoprotein; TG, triglycerides; HbA1C, hemoglobin A1c; WC, waist circumference; W/H, waist-to-hip ratio; HOMA-IR, homeostasis model assessment of insulin resistance; M, mean; SD, standard deviation, QR: Inter quartile range, SD: Standard deviation, χ^2^: Chi square test, H: H for Kruskal Wallis test, p: p value for comparing between the three studied groups *: Statistically significant at p ≤ 0.05

### Metabolic risk abnormalities in NAFLD/MAFLD/MASLD

**Fig. 2:**
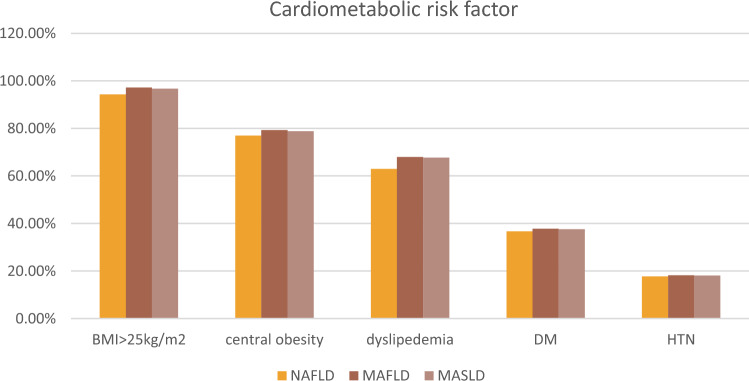
Cardio-metabolic risk factors of the studied groups

For NAFLD patients, Elevated BMI > 25 kg/m2 emerged as the predominant metabolic disorder factor (94.3%), then 77% had central obesity, while 66% had dyslipidemia and 36.7% had diabetes mellitus (DM). Whereas hypertension (HTN) was the least common metabolic disorder factor (17.7%). As to MAFLD patients, 347 (97.2%) had a BMI ≥ 25 kg/m2, 192 (53.78%) had HOMA-IR ≥ 2.5, 165 (46.21%) had elevated triglyceride levels, 135 (37.8%) were found to have type 2 diabetes, and 65 (18.2%) were hypertensive. For MASLD patients, the most common cardio-metabolic risk factor (CMRF) criteria were BMI ≥ 25 kg/m2 (*n* = 347, 96.7%), followed by (*n* = 135, 37.60%) were diabetic, (*n* = 65, 18.1%) were hypertensive, (*n* = 165, 46%) had elevated triglyceride levels. (*n* = 192, 53.48%) had HOMA-IR ≥ 2.5 (Fig. 2).

### Cardiovascular risk, and liver fibrosis and steatosis under different fatty liver disease

Non-invasive markers of hepatic fibrosis did not differ significantly, with median APRI, FIB-4, and NFS were around (0.042, 1.05, and −1.2) respectively. Similarly, were the results of non-invasive markers of hepatic steatosis with median HIS and FLI were around (46, and 91) respectively. Regarding to 10-year ASCVD risk factor revealed non statistically significant difference with median around 0.026 (Table 3).

**Table 3:** Cardiovascular risk, and hepatic fibrosis and steatosis of the studied population

	**NAFLD** **(n = 369)**	**MAFLD** **(n = 357)**	**MASLD** **(n = 359)**	**Test of Sig**	**P**
**CVD Risk**					
Low	241 (65.3%)	229 (64.1%)	231 (64.3%)	χ^2^ = 0.103	1.000
Borderline	46 (12.5%)	46 (12.9%)	46 (12.8%)		
Intermediate	57 (15.5%)	57 (16.0%)	57 (15.9%)		
High	25 (6.7%)	25 (7.0%)	25 (7.0%)		
**ASCVD**					
Min. – Max	0.001 – 0.589	0.001 – 0.589	0.001 – 0.589	H = 0.792	0.673
Mean ± SD	0.057 ± 0.081	0.061 ± 0.086	0.059 ± 0.084		
Median (IQR)	0.025(0.008 – 0.068)	0.027(0.009 – 0.070)	0.026(0.009 – 0.070)		
**APRI**					
Min. – Max	0.09 – 2.96	0.09 – 2.96	0.09 – 2.96	H = 0.024	0.988
Mean ± SD	0.53 ± 0.35	0.53 ± 0.35	0.53 ± 0.35		
Median (IQR)	0.42 (0.31 – 0.62)	0.42 (0.31 – 0.62)	0.42 (0.31 – 0.62)		
**FIB-4**					
Min. – Max	0.26 – 7.74	0.26 – 7.74	0.26 – 7.74	H = 0.097	0.953
Median (IQR)	1.05 (0.74 – 1.47)	1.07 (0.76 – 1.47)	1.07 (0.76 – 1.47)		
**HIS**					
Min. – Max	25.95 – 85.73	25.96 – 85.73	25.96 – 85.73	H = 0.500	0.779
Mean ± SD	46.49 ± 8.08	46.92 ± 7.81	46.78 ± 7.95		
Median (IQR)	45.93(41.26 –50.91)	46.23(41.83 –51.13)	46.19(41.68 –51.11)		
**FLI**					
Min. – Max	7 – 100	7 – 100	7 – 100	H = 0.285	0.867
Mean ± SD	84.4 ± 15.9	85.1 ± 15.3	84.6 ± 16.2		
Median (IQR)	91 (77 – 96)	91 (78 – 96)	91 (78 – 96)		
**NFS**					
Min. – Max	−5.39 – 2.82	−5.22 – 2.82	−5.22 – 2.82	H = 0.313	0.855
Mean ± SD	−1.22 ± 1.47	−1.16 ± 1.44	−1.17 ± 1.45		
Median (IQR)	−1.20 (−2.28 – −0.23)	−1.15 (−2.18 – −0.20)	−1.15 (−2.20 – −0.21)		

CVD, cardiovascular disease; ASCVD, atheroscelotic cardiovascular disease;APRI, AST-to-platelet ratio index; FIB-4, fibrosis-4 score; HIS, hepatic steatosis index; FLI, fatty liver index; NFS, NAFLD fibrosis score; M, mean; SD, standard deviation; OR, odds ratio; CI, confidence interval, QR: Inter quartile range, SD: Standard deviation, χ^2^: Chi square test, H: H for Kruskal Wallis test, p: p value for comparing between the three studied groups *: Statistically significant at p ≤ 0.05

### Characteristics of pure NAFLD cases

Out of 10 pure NAFLD patients, 90% was male (90.0%), with only 10.0% being female. The mean age was 35.4 years, with a narrow range (31.0–41.0 years), suggesting a relatively homogenous age group. The mean BMI was 24.18 ± 0.47 kg/m^2^, liver enzymes (AST, ALT, and GGT) show mild elevation, with GGT levels being the most variable (22.0–71.0 U/L), INR values are within the normal range, Lipid parameters reveal slightly elevated LDL (mean 102.5 ± 42.87 mg/dL), while total cholesterol, HDL, and triglycerides remain within acceptable ranges. HbA1C levels (mean 5.06 ± 0.25%) indicate normoglycemia. HOMA-IR (mean 3.07 ± 0.33) suggests mild insulin resistance, a characteristic finding in NAFLD. Waist circumference (mean 87.6 ± 2.95 cm) and waist-to-hip ratio (mean 0.88 ± 0.03). Steatosis indices such as the Hepatic Steatosis Index (HSI) and Fatty Liver Index (FLI) exhibit moderate elevations, supporting the diagnosis of NAFLD. Non-invasive fibrosis markers (APRI, FIB-4, ALBI and NFS) indicate low fibrosis risk, as expected in a cohort with early or pure NAFLD. The ALBI score remains in the negative range, reflecting well-preserved liver function (Table 4).

**Table 4:** Characteristics of pure NAFLD cases (n = 10)

	**No. (%)**	**Min. – Max**	**Mean ± SD**	**Median (IQR)**
**Gender**				
Male	9 (90.0%)			
Female	1 (10.0%)			
**Age (years)**		31.0 – 41.0	35.40 ± 2.91	35.0 (34.0 – 37.0)
**BMI (kg/m**^**2**^**)**		23.14 – 24.9	24.18 ± 0.47	24.2 (24 – 24.5)
**AST**		12.0 – 45.0	30.30 ± 9.75	32.50 (24.8 – 36.8)
**ALT**		23.0 – 46.0	35.80 ± 6.39	36.0 (33.0 – 41.0)
**GGT**		22.0 – 71.0	45.40 ± 16.0	44.0 (35.0 – 54.0)
**INR**		0.93 – 1.10	1.01 ± 0.05	1.0 (0.98 – 1.04)
**Platelet**		153.0 – 398.0	243.5 ± 70.71	229.0 (210.0 – 280.0)
**T. cholesterol**		147.0 – 198.0	158.7 ± 14.34	155.0 (159.0 – 159.0)
**HDL**		41.0 – 56.0	44.70 ± 4.24	44.0 (42.3 – 45.0)
**LDL**		71.40 – 222.2	102.5 ± 42.87	92.20 (87.60 – 94.40)
**TG**		83.0 – 133.0	104.8 ± 14.16	101.5 (98.0 – 106.0)
**HbA1C**		4.80 – 5.6	5.06 ± 0.25	5.0 (4.90 – 5.34)
**ASCVD**		0.003 – 0.008	0.006 ± 0.002	0.007 (0.005 – 0.007)
**Waist circumference**		80.0 – 90.0	87.6 ± 2.95	88.0 (87.0 – 90.0)
**Waist/hip**		0.80 – 0.90	0.88 ± 0.03	0.89 (0.85 – 0.90)
**HOMA-IR**		2.46 – 3.68	3.07 ± 0.33	3.06 (2.89 – 3.16)
**QUIKI**		0.32 – 0.33	0.32 ± 0.01	0.32 (0.32 – 0.33)
**APRI**		0.24 – 1.29	0.48 ± 0.33	0.37 (0.28 – 0.51)
**ALBI**		−4.08 – −3.23	−3.73 ± 0.25	−3.83 (−3.83 – −3.57)
**FIB-4**		0.45 – 2.60	0.94 ± 0.69	0.66 (0.50 – 0.92)
**Hepatic steatosis index**		25.95 – 40.70	34.35 ± 4.29	34.36 (32.64 – 36.37)
**FLI**		20.0 – 79.0	43.20 ± 18.11	41.0 (34.0 – 51.0)
**NAFLD fibrosis score**		−5.39 – −0.93	−3.16 ± 1.24	−3.30 (−3.82 – −2.45)

AST, aspartate aminotransferase; ALT, alanine aminotransferase; GGT, gamma-glutamyl transferase; INR,

international normalized ratio; HDL, high-density lipoprotein; LDL, low-density lipoprotein; TG, triglycerides; HbA1C, hemoglobin A1c; WC, waist circumference; W/H, waist-to-hip ratio; HOMA-IR, homeostasis model assessment of insulin resistance; M, mean; SD, standard deviation, QR: Inter quartile range, SD: Standard deviation, χ^2^: Chi-square test, H: H for Kruskal Wallis test, p: p-value for comparing between the three studied groups *: Statistically significant at p ≤ 0.05

## Discussion

MAFLD and MASLD represent significant advancements over the NAFLD framework, with both placing a greater emphasis on metabolic dysfunction. However, their approach differs in several ways, **MAFLD** requires hepatic steatosis, overweight/obesity, type 2 diabetes mellitus, and two of seven metabolic risk factors, making it more comprehensive but slightly complex. **MASLD**, by requiring only one of five cardiometabolic risk factors, simplifies the diagnostic process, potentially enhancing applicability in clinical and research settings. This distinction has implications for patient classification and could influence clinical decision-making, particularly in populations with borderline metabolic risk profiles.

The endorsement of MAFLD by over 80 professional societies, as noted in Pan et al., reflects its global acceptance as a practical framework for identifying metabolically at-risk populations. Meanwhile, MASLD, as proposed by a multi-society consensus, aims to unify terminologies across regions, facilitating broader acceptance and integration into practice (15]. This shift allows for earlier identification of high-risk patients who may benefit from targeted interventions, thereby promoting a more personalized approach to management that integrates liver health with overall metabolic health. Additionally, the distinction between MAFLD and MASLD guides clinical management, with MAFLD patients often requiring intensive lifestyle changes, metabolic-targeted therapies, and close monitoring for liver complications. In contrast, MASLD highlights the need for proactive management of cardiovascular and metabolic risks, even in patients without obvious metabolic dysfunction [15–17].

The current study provided an insightful evaluation of the application of the newly proposed diagnostic criteria for MAFLD and MASLD in a cohort of patients initially diagnosed with NAFLD. Our findings elucidated key differences and overlaps between the traditional NAFLD diagnosis and the more recently adopted MAFLD and MASLD frameworks, with significant implications for clinical practice and disease management.

In this study, 97.55% of NAFLD patients met the MASLD criteria, and 97.01% met the MAFLD criteria. This results suggested that while both MAFLD and MASLD criteria identify patient groups comparable to NAFLD, the MASLD framework appears to encompass a broader spectrum, accommodating a larger number of patients.

This finding aligns with previous studies demonstrating substantial overlap between these categories with approximate profiles. For instance, **Eslam et al. (2020)** showed that MAFLD captures most patients diagnosed with NAFLD, due to its broader criteria that incorporate metabolic risk factors such as type 2 diabetes, obesity, and dyslipidemia [18]. Similarly, MASLD, the newly proposed nomenclature, also includes patients traditionally classified under NAFLD but considered cardio-metabolic factors, echoing findings from the current analysis. Regarding the relatively strict and complex MAFLD definition, MASLD simplified the number of metabolic risk factors. This phenomenon was also observed in a previous study from **Hong Kong cohort 2024** (MASLD: 26.7% vs. MAFLD: 25.9%) [19].

Considerably, hormonal imbalances, such as insulin resistance, altered adipokines, and inflammatory markers like highly sensitive-reactive protein (hs-CRP), IL-6, and TNF-α, are pivotal in fatty liver pathogenesis and cardiovascular risk. While these markers offer insights into systemic inflammation and metabolic dysfunction, the MASLD criteria exclude CRP and HOMA-IR for broader applicability, focusing on universally recognized risk factors like obesity, type 2 diabetes, and dyslipidaemia. However, this approach may overlook disease heterogeneity and limit precise patient stratification [20].

Accordingly, a small subset of NAFLD patients (n = 10) were excluded from MAFLD/MASLD frameworks despite potentially significant liver pathology because they were lean and lacked 2 of 7 metabolic risk factors (MAFLD) or 1 of 5 cardiometabolic risk factors (MASLD). This supports the notion that lean NAFLD is a distinct entity, as discussed in studies like **Ye et al. (2020),** which highlighted that lean NAFLD patients may have a different pathophysiological profile than those with metabolic risk factors. These patients tend to be younger, have lower BMI, and have lower liver enzyme levels, consistent with the characteristics of the lean NAFLD group in this study [21]. However, the relatively small number of such cases suggests that lean NAFLD represents only a minority within the broader spectrum of fatty liver disease.

Despite being lean, these patients are not necessarily at lower risk for liver disease progression as they can present with significant histological severity, including fibrosis, inflammation, and increased mortality risk. Comparing lean NAFLD with metabolically abnormal patients under MAFLD or MASLD can provide insights into unique pathophysiological pathways driving liver disease in non-obese populations. Lean NAFLD cases may harbor subtle metabolic abnormalities not captured by routine criteria (e.g., adipokine dysregulation, ectopic fat, or mitochondrial dysfunction) [22, 23]. Both MAFLD and MASLD frameworks exclude these cases due to a lack of qualifying metabolic dysfunction. This exclusion risks delaying diagnosis and missing opportunities for early lifestyle or pharmacological interventions. Therefore, it is imperative to emphasize the necessity of improving these frameworks to incorporate high-risk but non-obese phenotypes. Refining MAFLD and MASLD frameworks might be through the incorporation of additional diagnostic criteria for lean NAFLD, such as liver stiffness measurement, inflammatory biomarkers, or advanced imaging for ectopic fat. Alternatively, subcategorization of lean NAFLD as a distinct phenotype within these frameworks to ensure appropriate inclusion and management [20].

Although the number of such patients was limited, future studies should focus on characterizing lean NAFLD/MAFLD/MASLD populations to uncover their specific risk factors, natural history, and therapeutic needs.

Notably, in addition to the 10 lean cases, MAFLD's capacity to identify cases was further constrained, as it failed to recognize two additional cases with steatosis and elevated high-density lipoprotein levels (HDL) which represent only one of the seven cardiometabolic criteria defined by MAFLD.

One of the main observations of the current study was the absence of statistically significant differences in the clinical features (including BMI, waist circumference, liver enzymes, lipid profile, blood glucose, HOMA-IR, ASCVD, FIB4, APRI, NFS, HIS and FLI) between the three groups (p > 0.05), which is an important insight in understanding the overlap between these disease entities. This is consistent with the findings from **Song et al. and Kaya et al. **who demonstrated negligible differences between the various definitions NAFLD, MAFLD and MASLD [24, 25].

Both frameworks (MAFLD and MASLD) show promise in identifying high-risk groups for liver fibrosis and cardiovascular outcomes. Studies such as **Zhou et al. (2024) and Jiang et al. (2024)** emphasized the utility of these criteria in predicting disease progression and comorbidities. However, it remains unclear whether the simplified MASLD criteria offer the same precision as MAFLD in stratifying risk, particularly in lean patients or those with subtle metabolic alterations [19, 26].

The study population predominantly consisted of overweight or obese individuals, with 97.2% of MAFLD patients and 96.7% of MASLD patients having a BMI ≥ 25 kg/m^2^. This is in agreement with previous research that highlights obesity as a dominant risk factor in fatty liver diseases across the NAFLD, MAFLD, and MASLD spectrum. The slight variation in the proportions of patients with metabolic risk factors (such as diabetes, hypertension, and dyslipidemia) suggests that the new definitions of MAFLD and MASLD might identify a slightly broader cohort than the original NAFLD definition, especially regarding lean patients. However, the overlap is significant, suggesting that the diagnostic criteria are mostly aligned in capturing metabolically unhealthy liver disease.

Among MAFLD and MASLD patients, the prevalence of type 2 diabetes (37.8% and 37.6%, respectively) and hypertension (18.2% and 18.1%, respectively) were nearly identical. This further demonstrates the substantial overlap in patient profiles between these categories, consistent with findings from multiple studies. For example, a large cohort study by Kim et al. (2021) reported similar rates of metabolic comorbidities in patients diagnosed with MAFLD versus NAFLD, reinforcing the idea that MAFLD and MASLD might serve as improved clinical frameworks to identify patients with increased cardiometabolic risk [15].

The study found no significant differences in APRI, FIB-4, or the NAFLD fibrosis score across the three groups. This is consistent with research indicating that fibrosis severity is largely driven by metabolic risk factors, which are common across NAFLD, MAFLD, and MASLD patients. Studies such as those by Younossi et al. (2019) also report that fibrosis progression in fatty liver disease is more dependent on metabolic dysfunction (e.g., diabetes, hypertension) than on the specific diagnostic criteria used. Thus, the lack of difference in fibrosis markers between the groups supports the notion that fibrosis severity is likely more influenced by metabolic risk burden rather than the specific categorization of the liver disease [27].

The study found no significant differences in lipid profiles (TC, LDL-C, HDL-C, and triglycerides) between NAFLD, MAFLD, and MASLD patients. Likewise, the risk of cardiovascular disease (CVD), evaluated using ASCVD scores, was comparable across the groups. This mirrors findings from studies like Targher et al. (2021), which emphasized that the presence of metabolic risk factors such as diabetes and dyslipidemia are major contributors to cardiovascular risk in liver disease patients, regardless of their specific classification as NAFLD, MAFLD, or MASLD. The similar CVD risk profiles in the present study suggest that the reclassification of NAFLD into MAFLD or MASLD does not dramatically alter the cardiovascular risk burden of these patients [28].

The strengths of this study include its large sample size of 369 patients, which enhances the reliability of the findings, and its novel approach of comparing three diagnostic criteria NAFLD, MAFLD, and MASLD while assessing comprehensive metabolic, cardiovascular, and liver fibrosis markers. This real-world setting reflects the clinical challenges of diagnosing and managing fatty liver disease with diverse metabolic profiles. However, the study is limited by its single-center, cross-sectional design, restricting its generalizability and causality assessment. The cross-sectional nature of this study limits the ability to establish causality or evaluate the long-term implications of reclassification on patient outcomes. As suggested by Targher et al. (2021), longitudinal studies are essential to determine whether these new frameworks improve clinical outcomes, such as progression to advanced fibrosis, cardiovascular events, or overall mortality. Such studies will also provide insights into the temporal stability of the MAFLD and MASLD criteria in different clinical settings.

Conclusively, this study underscores the utility of the MAFLD and MASLD diagnostic criteria in classifying NAFLD patients while highlighting their strengths in encompassing metabolic risk factors. MASLD criteria slightly broaden patient inclusion compared to MAFLD, enhancing clinical applicability. However, a subset of lean NAFLD patients with potential metabolic and liver disease risks remains uncaptured by these frameworks, necessitating further refinement. Future research should focus on integrating novel biomarkers and personalized diagnostic approaches to address disease heterogeneity and improve patient stratification and outcomes.

## Data Availability

‘Data is available at the corresponding author upon request”.

## Abbreviations

HTN: Hypertension
DM: Diabetes mellitus
BMI: Body mass index
AST: Aspartate aminotransferase
ALT: Alanine aminotransferase
GGT: Gamma-glutamyl transferase
INR: International normalized ratio
HDL: High-density lipoprotein
LDL: Low-density lipoprotein
TG: Triglycerides
HbA1C: Hemoglobin A1c
WC: Waist circumference
W/H: Waist-to-hip ratio
HOMA-IR: Homeostasis model assessment of insulin resistance
QUIKI: Quantitative insulin sensitivity check index
APRI: AST-to-platelet ratio index
ALBI: Albumin-bilirubin index
FIB-4: Fibrosis-4 score
HIS: Hepatic steatosis index
FLI: Fatty liver index
NFS: NAFLD fibrosis score
M: Mean
SD: Standard Deviation
OR: Odds ratio
CI: Confidence Interval
QR: Inter quartile range
SD: Standard deviation
